# Targeting HMGB1 by ethyl pyruvate ameliorates systemic lupus erythematosus and reverses the senescent phenotype of bone marrow-mesenchymal stem cells

**DOI:** 10.18632/aging.102052

**Published:** 2019-07-14

**Authors:** Juan Ji, Ting Fu, Chen Dong, Wenyan Zhu, Junling Yang, Xiaoli Kong, Zhongyuan Zhang, Yanfeng Bao, Rui Zhao, Xinyu Ge, Xiaoqi Sha, Zhimin Lu, Jing Li, Zhifeng Gu

**Affiliations:** 1 Department of Rheumatology, Affiliated Hospital of Nantong University, Nantong, Jiangsu 22600, P.R. China; 2 Research Center of Clinical Medicine, Affiliated Hospital of Nantong University, Nantong, Jiangsu 226001, P.R. China; 3 Department of Medical Cosmetology, Affiliated Hospital of Nantong University, Nantong, Jiangsu 226001, P.R. China

**Keywords:** systemic lupus erythematosus, mesenchymal stem cells, senescence, HMGB1, inflammation

## Abstract

Systemic lupus erythematosus (SLE) is a chronic autoimmune disease involving multiple organs and systems. Mesenchymal stem cells (MSCs) from SLE patients have demonstrated defects such as impaired growth, senescence phenotype and immunomodulatory functions. Some studies have suggested the close connection between inflammation microenvironment and cellular senescence. In the current study, we detected cytokines levels in bone marrow supernatant by the quantitative proteomics analysis, and found the expression of HMGB1 was remarkably increased in bone marrow from SLE patients. Senescence associated-β-galactosidase (SA-β-gal) staining, F-actin staining and flow cytometry were used to detect the senescence of cells. After stimulation of HMGB1 in normal MSCs, the ratio of SA-β-gal positive in BM-MSCs was increased, the organization of cytoskeleton was disordered, and TLR4-NF-κB signaling was activated. Finally, Ethyl pyruvate (EP) (40 mg/kg and 100 mg/kg, three times a week), a high security HMGB1 inhibitor, was injected intraperitoneally to treat MRL/lpr mice for 8 weeks. We demonstrated that EP alleviated the clinical aspects of lupus nephritis and prolonged survival of MRL/lpr mice. In the meantime, EP reversed the senescent phenotype of BM-MSCs from MRL/lpr mice. HMGB1 could be a promising target in SLE patients, and might be one of the reasons of recurrence after MSCs transplantation.

## INTRODUCTION

Systemic lupus erythematosus (SLE), a chronic autoimmune disease, involves multiple organs and systems and influences patients on health and life severely [1]. With recent advances in understanding of the underlying pathology, utilizing targeted biological agents in SLE patients properly has resulted in a significant improvement in prognosis. Nonetheless, the search for new therapeutic strategies is relentless because of the occurrence of refractory disease [2]. Mesenchymal stem cells (MSCs) are identified as promising and alternative cells because of their self-renewal, pluripotent differentiation ability and low immunogenicity [3]. Previous study showed that allogenic MSC transplantation (MSCT) seemed to be a safe and effective therapeutic strategy in refractory SLE [4]. Further studies showed that syngeneic MSCT was ineffective [5]. Our and other group reported that BM-MSCs from SLE patients showed characteristics of senescence, appearing increased Senescence associated- β-galactosidase (SA-β-gal) activity, cell cycle arrest, disordered F-actin distribution and reduced ability of regulating Treg [6]. Reversing senescence of SLE MSCs pretreated with rapamycin *in vitro* could alleviate the clinical symptoms of lupus nephritis and prolong survival in MRL/lpr mice [7]. These studies revealed that senescent BM-MSCs might be associated with the pathogenesis of SLE. It reminded us to discover the senescence mechanism of BM-MSCs thoroughly.

Microenvironmental inflammatory factors may participate in the process of cellular senescence [8]. According to recent researches, a long list of proinflammatory cytokines, including tumor necrosis factor-α (TNF-α), interferon-γ (IFN-γ) and so on, are capable of inducing cellular senescence and bone defects in aging [9]. Our previous study showed that TNF-α had a negative impact on osteogenic differentiation of MSCs via the NF-κB signaling pathway [10]. Sun et al found that leptin and neutrophil activating protein 2, the top 2 upregulated factors in SLE serum, played a major role in promoting MSCs senescence by activating PI3K/Akt pathway [11]. It suggested that the senescence of BM-MSCs in SLE could be attributed to the bone marrow microenvironment inflammatory factors.

Therefore, our present work was to probe the key factor in bone-marrow microenvironment on senescence of BM-MSCs. We analyzed bone-marrow ingredients difference between SLE patients and healthy controls by proteomics. On this basis, we further discovered the effect of inflammation in bone-marrow microenvironment on senescence of BM-MSCs in SLE and the underlying mechanism. The study helps us to open new perspectives for MSCT treatment of SLE.

## RESULTS

### HMGB1 was elevated in bone marrow supernatant from SLE patients

**Figure 1:**
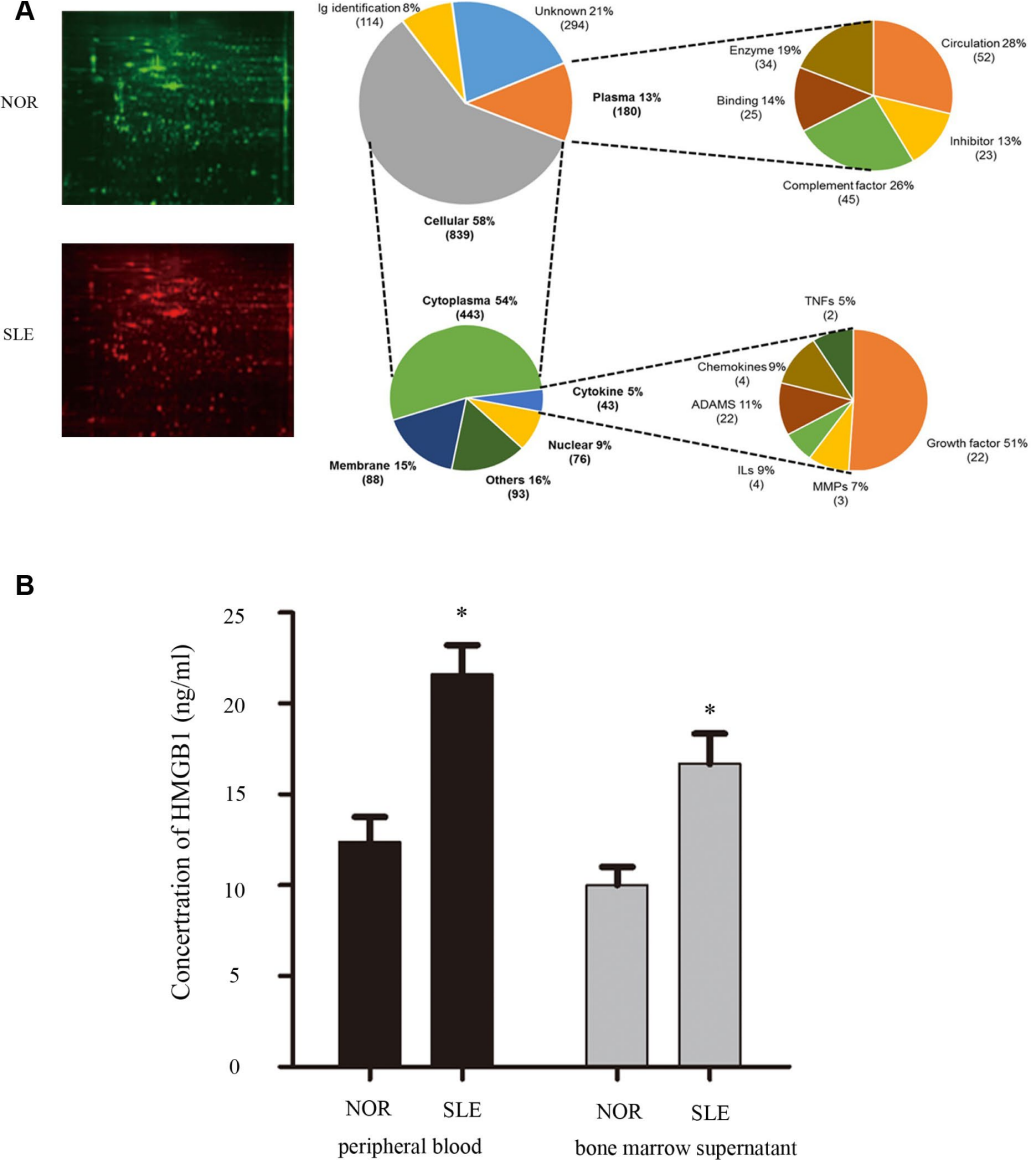
(**A**) We collected bone marrow supernatant from normal people and SLE patients. Bone marrow supernatant samples were analyzed using a proteomics approach. (**B**) Elisa showed that HMGB1 expression levels were high in bone marrow supernatant and serum of SLE patients. (Bar represents mean ± SD,*P < 0.05 compared with the normal group) (NOR=normal group, SLE=systemic lupus erythematosus patients group).

To discover the crucial factor in bone marrow microenvironment which was involved in MSCs senescence, we collected bone marrow supernatant from SLE patients and healthy controls. Our proteomics results showed that expressions of multiple inflammatory cytokines were abnormal in bone marrow supernatant of SLE patients, including HMGB1, IFN-γ, and so on (Figure 1A). To verify the data from proteomics results, bone marrow supernatant level of HMGB1 was measured by ELISA, and the elevated expression of HMGB1 was also detected in SLE patients, compared to the healthy control (Figure 1B).

### Bone marrow supernatant from SLE patients led MSCs to senescence via HMGB1/TLR4/NF-κB signaling pathway

**Figure 2:**
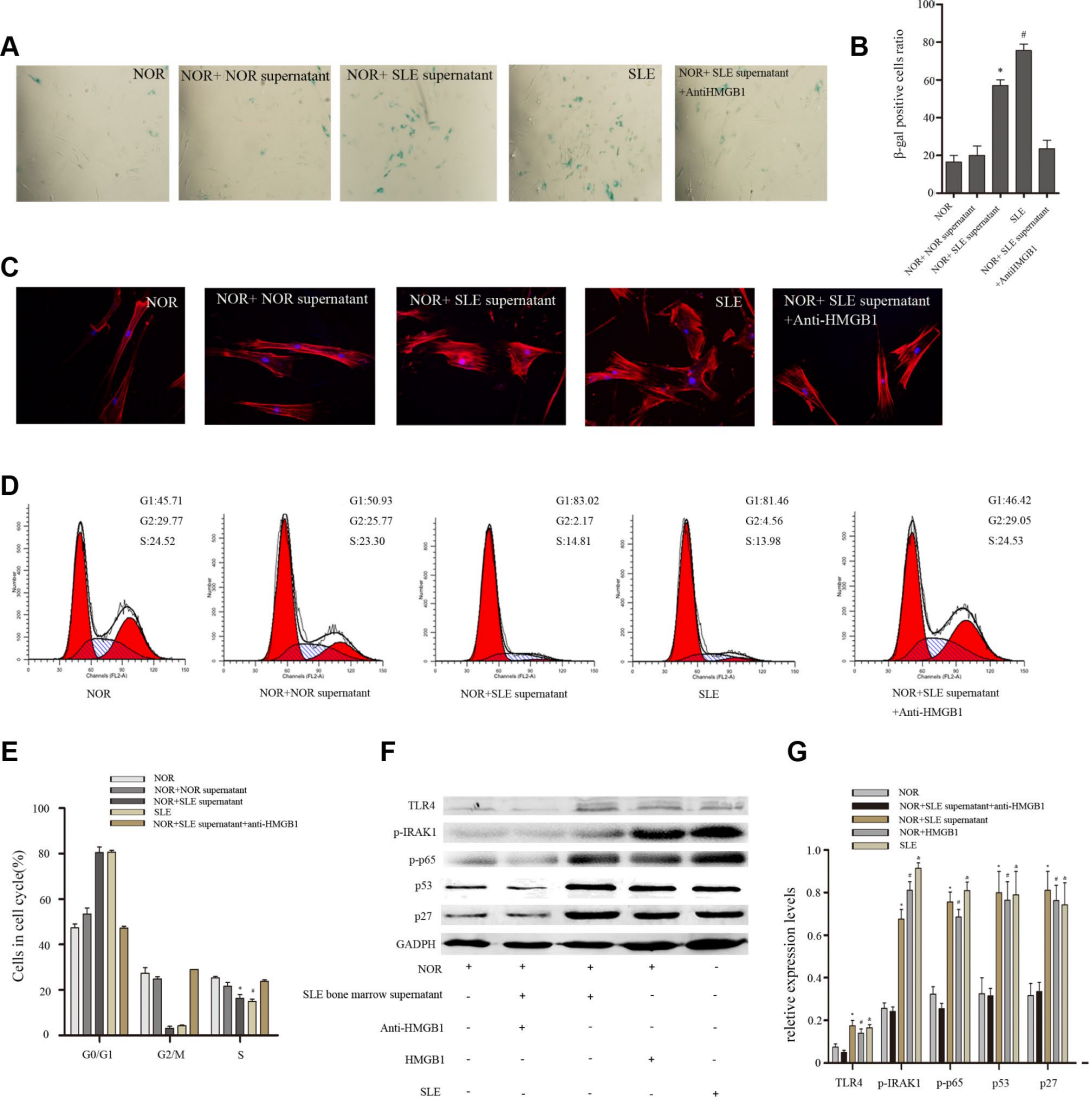
Normal BM-MSCs were treated with bone marrow supernatant from normal persons or SLE patients, or MSCs were cocultured with SLE bone marrow supernatant treated with anti-HMGB1 mAb. Five groups were analyzed. (**A**, **B**) BM-MSCs were fixed and stained with SA-β-gal. (**C**) MSCs were stained by fluorescein isothiocyanate-conjugated phalloidin. The distribution of F-actin was disordered after treatment with bone marrow supernatant from SLE patients by Immunofluorescence. (**D**, **E**) Cell viability was assessed by flow cytometry analysis. (**F**, **G**) Normal BM-MSCs were treated with SLE bone marrow supernatant or 100 ng/ml HMGB1, or MSCs were cocultured with SLE bone marrow supernatant treated with anti-HMGB1 mAb. Five groups were analyzed. TLR4, p-IRAK1, p-p65, p53 and p27 expressions were analyzed by western blot. GAPDH was used as the internal control. (Bar represents mean ± SD,*P < 0.05 compared with the normal group, #P < 0.05 compared with the normal group, &P < 0.05 compared with the normal group) (NOR=normal group, SLE=systemic lupus erythematosus patients group).

To further determine the effects of SLE bone marrow microenvironment on senescence of BM-MSCs, bone marrow supernatant was collected and used to stimulate BM-MSCs. Our results showed there were more SA-β-gal-positive cells in BM-MSCs when treated with bone marrow supernatant from SLE patients, interestingly, which could be reversed by a neutralizing anti-HMGB1 monoclonal antibody (Figure 2A, 2B). Furthermore, Immunofluorescence analysis showed that the distribution of F-actin in BM-MSCs was disordered after treatment with bone marrow supernatant from SLE patients (Figure 2C). The G1 phase was induced when normal BM-MSCs were treated with bone marrow supernatant from SLE patients by cell cycle analysis (Figure 2D, 2E). Western blotting analysis showed that expressions of cell cycle relation proteins, p53 and p27 were high in BM-MSCs treated by SLE bone marrow supernatant (Figure 2F). Anti-HMGB1 mAb treatment in SLE bone marrow supernatant did not lead to any significant changes in F-actin distribution and G1 phase. TLR4, one of HMGB1 receptors, can modulate MSCs function, trigger intracellular signaling pathways, and lead to the induction of inflammatory cytokines [12]. To discover the mechanism of bone marrow supernatant in the senescence of MSCs from SLE patients, we examined the expression of components of TLR4/NF-κB pathway. As shown in Figure 2F, bone marrow supernatant from SLE patients promoted the expression of TLR4 and its proteins expressed by its downstream regulated genes, p-IRAK1 and p-p65 (Figure 2F, 2G). These results confirmed that inflammatory microenvironment played facilitation to senescence of BM-MSCs, and HMGB1 had great significance.

### HMGB1 accelerated the senescence of normal BMMSCs by activating TLR4/ NF-κB signaling pathway

**Figure 3:**
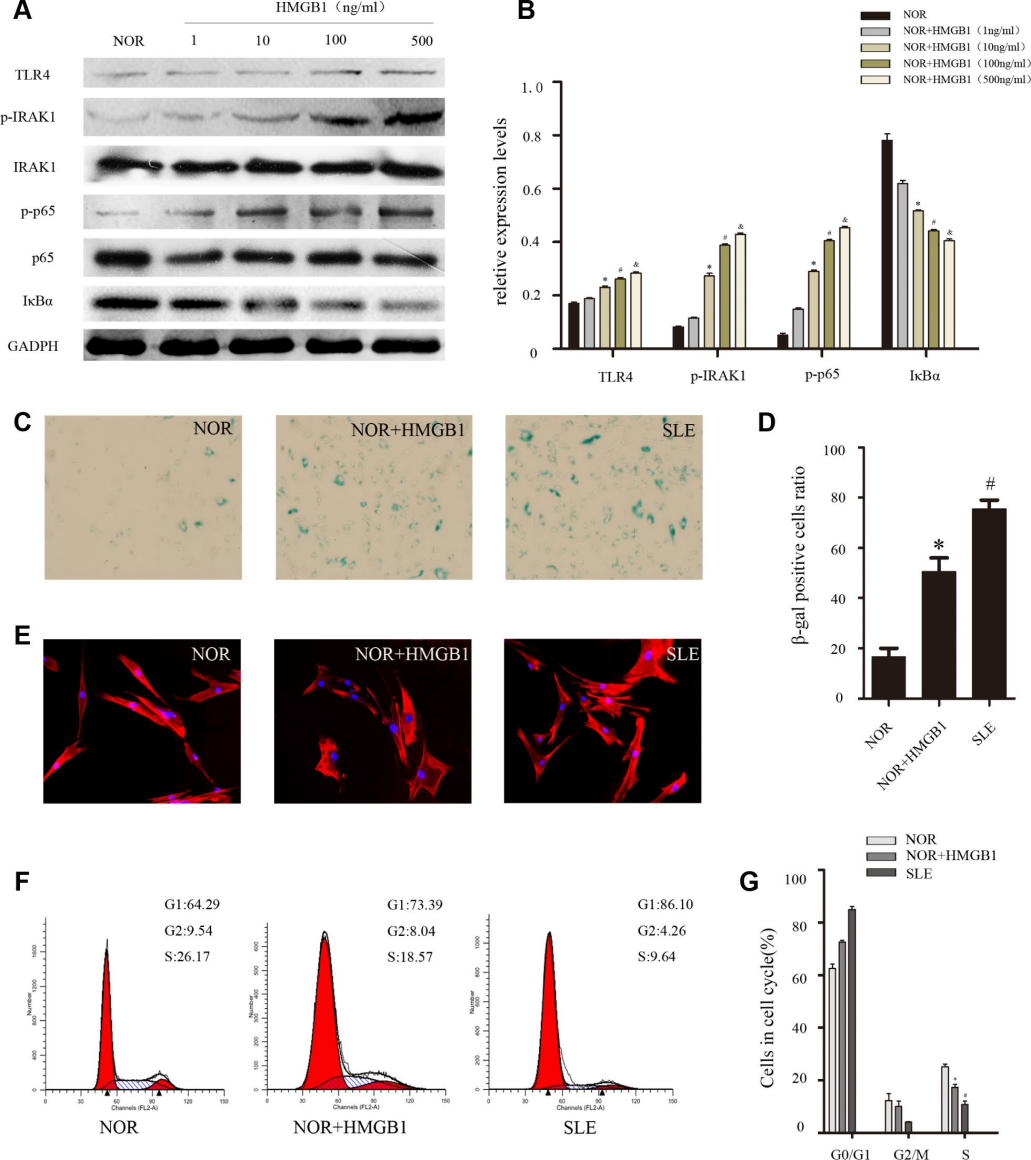
NOR MSCs were cultured at the different concentration (1,10,100,500ng/ml) of HMGB1 for 48 h. (**A**, **B)** The expressions of TLR4, p-IRAK1, p-p65 and IκBa in MSCs were determined by western blot analysis. GAPDH was used as the internal control. (**C**, **D**) BM-MSCs were fixed and stained with SA-β-gal. (E)The distribution of F-actin was disordered after treatment with exogenous HMGB1 by Immunofluorescence. (**F**, **G**) Cell viability was assessed by flow cytometry analysis. (Bar represents mean ± SD,*P < 0.05 compared with the normal group, #P < 0.05 compared with the normal group, &P < 0.05 compared with the normal group) (NOR=normal group, SLE=systemic lupus erythematosus patients group).

To further confirm the role of HMGB1 in the senescence of MSCs from SLE patients, different concentrations of HMGB1(1,10,100,500ng/ml) were used to stimulate BM-MSCs, which resulted in the activation of TLR4/NF-κB signaling pathway and the senescence of BM-MSCs. We discovered that HMGB1 promoted TLR4 expression at a concentration of 10-100 ng/ml and achieved maximal effects at doses of 100-500 ng/ml (Figure 3A, 3B). Thus, 48 h of treatment with 100 ng/ml of HMGB1 was used in the subsequent experiments. Then, we explored the effects of HMGB1 on the senescence of BM-MSCs from SLE patients and found that there were more SA-β-gal-positive cells in the BM-MSCs from normal persons when treated with HMGB1 (Figure 3C, 3D). Furthermore, after treatment with HMGB1, the distribution of Factin in BM-MSCs was disordered (Figure 3E). In addition, G1 phase was induced via cell cycle analysis (Figure 3F, 3G). Our results showed that HMGB1 could activate the expression of TLR4/NF-κB signaling and promoted cellular senescence in normal BM-MSCs in vitro, which further implied that the TLR4/NF-κB signaling pathway may play an essential role in the senescence of SLE BM-MSCs.

### TLR4 signaling pathway participated in the senescence of BM-MSCs from SLE patients

**Figure 4:**
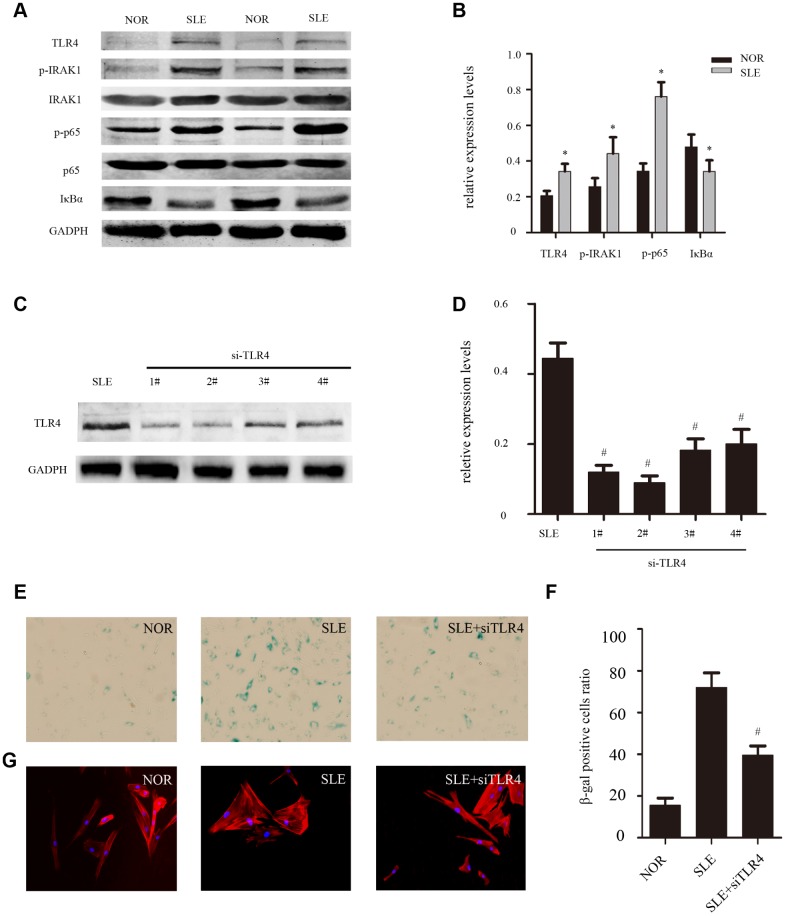
(**A**, **B**) The expression of TLR4, p-IRAK1, p-p65 and IκBa from SLE compared with normal group were determined by western blot analysis. GAPDH was used as the internal control. (**C**, **D**) MSCs were depleted of TLR4 by RNAi. The second one was chosen as the best siRNA by western blotting. (**E**, **F**) MSCs were fixed and stained for SA-β-gal. The number of SA-β-gal-positive cells obviously decreased among treated SLE MSCs in comparison with untreated group. (**G**) MSCs were stained by fluorescein isothiocyanate-conjugated phalloidin. The disordered distribution of F-actin was reversed in siTLR4-treated MSCs. (Bar represents mean ± SD,*P < 0.05 compared with the normal group, #P < 0.05 compared with the SLE group,) (NOR=normal group, SLE=systemic lupus erythematosus patients group).

Previous studies and our experiment results have shown that SLE MSCs exhibited signs of senescence [7]. To further determine the effects of the TLR4/NF-κB signaling pathway on MSCs senescence. We next studied the expression of components of the TLR4 signaling pathway in BM-MSCs by western blotting. The results showed that expressions of TLR4, p-IRAK1 and p-p65 were clearly increased in SLE BM-MSCs compared to that in normal persons, and degradation of IκBα was induced in SLE BM-MSCs (Figure 4A, 4B). Next, TLR4-siRNA was used to knockdown the expression of TLR4 in SLE BM-MSCs to clarify the correlation between TLR4 signaling and cellular senescence. SLE BM-MSCs were transfected with TLR4-siRNA#1–4 or control siRNA. After 48 h, the levels of TLR4 were evaluated by western blot analysis. It was found that TLR4-siRNA#2 significantly knocked down the expression of TLR4 compared with the control siRNA (Figure 4C, 4D). Furthermore, the ratio of SA-β-gal-positive cells (Figure 4E, 4F) and the distribution of F-actin (Figure 4G) in BMMSCs from SLE patients was effectively reversed after treatment with TLR4-siRNA. These results further implied that. the TLR4 signaling pathway might play an important role in the senescence of SLE BM-MSCs

### EP improved the survival rate and lupus nephritis by inhibiting the expression of HMGB1 in MRL/lpr mice

**Figure 5:**
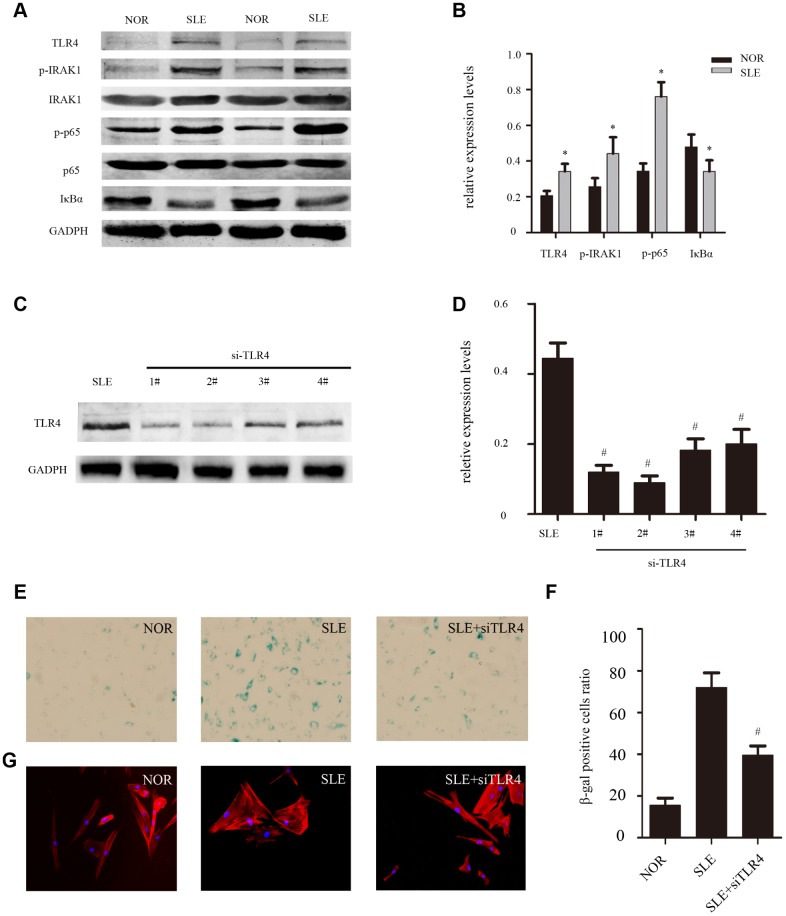
(**A**) 30 MRL/lpr mice were divided into three groups: MRL/lpr mice treated with normal saline, Ep-40mg/kg-treated MRL/lpr mice and Ep-100mg/kg -treated MRL/lpr mice. Ep was injected intraperitoneally to treat MRL/lpr mice aged 14 weeks for 8 weeks. (**B**) The expressions of HMGB1 in serum of MRL/lpr mice were examined by Elisa. (**C**) Survival curves observed that the survival rate of Ep-40mg/kg group and Ep-100mg/kg group was higher than that of control group. (**D**) Three groups MRL/lpr mice were weighed one time two weeks. (**E**) 24-hours urinary protein was measured by coomassie brilliant blue method. (**F**) HE-staining showed that renal pathological changes of MRL/lpr mice were significant, including glomerular sclerosis, mesangial cell proliferation, matrix widened, and lymphocytes infiltrating the interstitium. However, histopathological changes of other groups were alleviated. (Bar represents mean ± SD,*P < 0.05 compared with the control group, #P < 0.05 compared with the control group) (Con=Control group).

Previous study revealed that HMGB1 inhibition by a specific antibody could ameliorate albuminuria in MRL/lpr lupus-prone mice [13]. EP treatment significantly reduced circulating levels of HMGB1 in animals models and had protective effect [14]. Therefore, to further determine the effect of EP on MRL/lpr mice, mice were treated with EP according to the experimental design (Figure 5A). As expected, the expression of HMGB1 declined in serum of Ep-100mg/kg group, and the expression of HMGB1 in serum of Ep-40mg/kg group decreased moderately (Figure 5B). The survival rates in Ep-100mg/kg group were higher than that in saline solution-treated group (Figure 5C). The weight of the mice in Ep-40mg/kg group and Ep-100mg/kg group gradually increased (Figure 5D). The 24-hours urinary protein in these two groups were lower than those in control group (Figure 5E). In terms of pathology, glomerular sclerosis and interstitial fibrosis were ameliorated in Ep-100mg/kg group (Figure 5F). These results demonstrated EP had a significant therapeutic effect on LN by inhibiting HMGB1 in MRL/lpr mice.

### EP improved Treg immune regulation and reversed the senescence of BM-MSCs via inhibiting TLR4/ NF-κB signaling pathway

**Figure 6:**
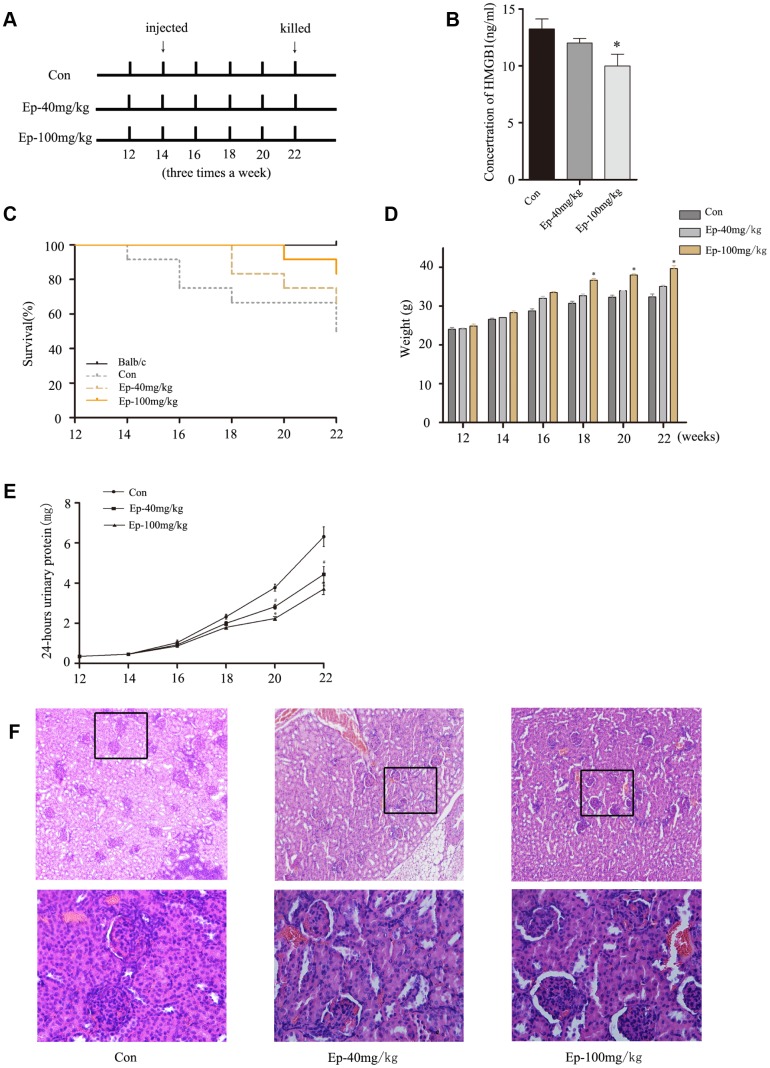
(**A**, **B**) the size comparison of spleen in three groups: MRL/lpr mice treated with normal saline, Ep-40mg/kg-treated MRL/lpr mice and Ep-100mg/kg -treated MRL/lpr mice. (**C**, **D**) Treg cell analysis in cells obtained from lymph nodes, spleen and peripheral blood. EP treatment upregulated the number of Treg cells. (**E**, **F**) BM-MSCs from MRL/lpr mice were isolated, then were fixed and stained for SA-β-gal. (**G**, **H**) Cell viability was assessed by flow cytometry analysis. (I, J) Expressions of TLR4, p-p65, and IκBa in MSCs from MRL/lpr mice, normal group and EP-treated group by Western blot analysis. (Bar represents mean ± SD,*P < 0.05 compared with the control group) (Con=Control group).

In the present study, we examined the influence of EP intraperitoneal injection on immune response of MRL/lpr mice, especially Treg immune. As shown in Figure 6A–6B, the size of spleen was significantly reduced in Ep-100mg/kg group. At the same time, EP treatment upregulated the number of Treg cells in peripheral blood, spleen, lymph nodes (Figure 6C, 6D). BM-MSCs from MRL/lpr mice showed senescent behavior, characterized by flattened and enlarged cell morphology, increased SA-β-gal activity, and cell cycle arrest. Interestingly, we observed decelerated cell senescence signs in BM-MSCs in Ep-100mg/kg group (Figure 6E, 6F). Cell-cycle data showed that BM-MSCs from MRL/lpr mice treated by EP displayed a significantly lower number of cells arrested in the G0/G1 phase compared with the control group (Figure 6G, 6H). Next, we investigated the expressions of TLR4, p-p65, and IκBa in MSCs from MRL/lpr mice, normal group and EP-treated group by Western blot analysis. We found higher levels of TLR4 and p-p65 in MSCs from MRL/lpr mice compared to the EP-treated group (Figure 6I, 6J). These results revealed that EP might be an effective therapeutic approach to SLE.

## DISCUSSION

**Figure 7:**
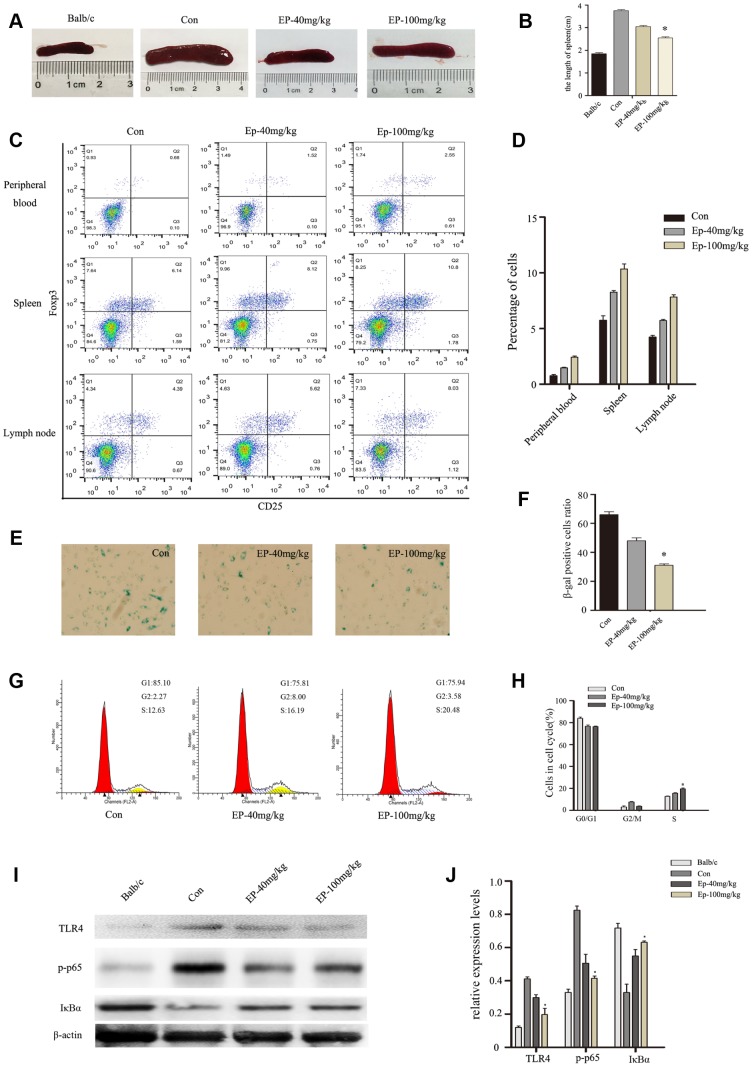
BM-MSCs and bone marrow supernatant were isolated from bone marrow of healthy donors and SLE patients. SLE bone marrow supernatant led MSCs to senescence via HMGB1/TLR4/NF-κB signaling pathway, HMGB1 had great significance. Ethyl pyruvate (EP), a high security HMGB1 inhibitor, was injected intraperitoneally to treat MRL/lpr mice aged 14 weeks for 8 weeks. EP alleviated the clinical aspects of lupus nephritis and prolonged survival of MRL/lpr mice, by reversing the senescent phenotype of BM-MSCs from MRL/lpr mice.

MSCs are pluripotent and imunoregulatory cells that are considered as a promising new treatment for autoimmune diseases [15]. Recently, a multicenter clinical trial showed MSCT resulted in effective clinical response in SLE patients. However, several patients experienced disease relapse after 6 months [16]. In this study, we firstly demonstrated that inflammatory factor HMGB1 in SLE bone marrow microenvironment could promote the senescence of MSCs via TLR4/NF-κB signaling pathway, and inhibiting HMGB1 by EP could improve lupus nephritis and reverse the senescence signs of MSCs (Figure 7). Therefore, higher expression of HMGB1 could partly explain why SLE patients needed to repeat MSCT after 6 months.

MSCs reside in a complex three-dimensional structure, which comprises hematopoietic stem cells, bone marrow, adipocytes, and so on [17]. The external environment could guide MSC biology, gradually deteriorate. Abnormity in the MSC microenvironment such as chronic inflammation could eventually lead to occurence of adverse manifestations, such as the accumulation of fat deposits in bone and muscles, or the change of hematopoiesis and autoimmunity [18]. Our previous study showed that repeated lipopolysaccharide(LPS) stimulation promoted cellular senescence in human dental pulp stem cells [19]. It was shown that the microenvironment into which MSCs were injected was critical and contained complex cellular interactions via soluble factors [20]. These studies indicated inflammatory cytokines played an essential role in the initiation and maintenance of cellular senescence, and were responsible for triggering MSCs senescence. Therefore, bone marrow supernatant of SLE patients and normal subjects was examined to represent the complicated bone marrow microenvironmental factor. There are a variety of elements in SLE bone marrow, including cytokines and chemokines. Furthermore, our results showed exogenous SLE bone marrow supernatant treatment could accelerate MSCs senescence. Among these raised cytokines, HMGB1 was significantly abnormal in bone marrow supernatant of SLE patients. These reminded us that HMGB1 in bone marrow microenvironment might play an important role in SLE MSCs senescence.

Recently, HMGB1 has been found to function as a potent proinflammatory cytokine [21]. Extracellular secreted HMGB1 stimulated senescence-associated secretory phenotype (SASP) through TLR4 signaling and NF-κB transcriptional activity [22]. HMGB-1, One of Damage-associated molecular patterns (DAMPs), inhibited the production of IFN-γ-induced the indoleamine-2,3-dioxygenase (IDO) by MSCs [23]. However, the influence of HMGB1 on cell function of MSCs remained controversial. Exogenous HMGB1 treatment and transfection with HMGB1 vectors promoted MSC migration and differentiate to endothelial cells [24]. HMGB-1 not only attracted MSCs, but also enhanced the MSCs proliferation and inhibited MSCs to produce IDO [25]. Another group found that HMGB1 protein inhibited the proliferation of human mesenchymal stem cells and promoted their migration and differentiation along osteoblastic pathway [26]. As described above, our study found there were more SA-β-gal-positive cells, disordered distribution of F-actin and cell cycle arrest when BM-MSCs were treated with bone marrow supernatant from SLE patients. Senescence signs could be reversed when HMGB1 was inhibited. We found that stimulation of exogenous HMGB1 also could promote senescence of MSCs. These results further suggested that HMGB1 played a functional role in senescence of MSCs.

Many inflammatory signaling pathways participated in aging and inflammation, such as NF-κB, mTOR, and MAPK [27, 28]. Various of evidences confirmed that the HMGB1-mediated inflammation could be mediated through TLR2, TLR4 and RAGE. HMGB1 could bind to TLR2, TLR4 and RAGE and result in downstream signaling pathway activation [29]. TLRs, especially TLR4, are highly expressed on MSCs and activation of TLR4 can significantly modulate the immunosuppressive and anti-inflammatory functions of MSCs [30]. HMGB1, released by activated platelets, could mediate inhibition of the cell death-dependent migratory response through regulating TLR4 on the MSCs [31]. HMGB1 and LPS activated TLR4-mediated NF-κB signaling pathway and proinflammatory response in human pericytes [32]. Other studies have found that pro-inflammatory cytokines(eg.IFN-γ, IL-1β) are up-regulated in SLE patients, which is associated with the activation of TLR4 [33]. Our results showed that exogenous HMGB1 stimulated TLR4/NF-κB transactivation activity in MSCs. We also found that TLR4/NF-κB activity was involved in senescence of MSCs. These provided strong evidence that HMGB1 enhanced senescence of MSCs by activating the TLR4/NF-κB signaling pathway.

**Table 1:** Details of 12 SLE patients.

**Patient**	**Age**	**Disease duration**	**Current treated**	**SLEDAI**
1	27	1 year	Pred 12.5mg/day	4
			HCQ 0.2g/day	
			CTX 0.4g/2 weeks	
2	43	19 years	Pred 10mg/day	8
			HCQ 0.2g/day	
			CTX 0.4g/2 weeks	
3	40	2 years	Pred 15mg/day	4
			HCQ 0.2g/day	
4	23	3 years	Pred 15mg/day	4
			HCQ 0.2g/day	
			MTX 5mg/1 week	
5	31	5 years	Pred 7.5mg/day	1
6	26	9 years	Pred 10mg/day	2
7	13	4 years	Pred 12.5mg/day	11
			HCQ 0.2g/day	
8	25	5 years	Pred 15mg/day	16
			HCQ 0.2g/day	
			CTX 0.4g/2 weeks	
9	25	8 years	Pred 10mg/day	8
			HCQ 0.2g/day	
10	31	6 years	Pred 15mg/day	8
			HCQ 0.2g/day	
11	35	7 years	Pred 15mg/day	10
			HCQ 0.2g/day	
12	23	2 years	none	5

Several new strategies have been developed to target some specific activation pathways relevant to the pathogenesis of SLE. For example, rapamycin treatment could reduce mTORC1 and enhance mTORC2 activities of lupus, so it has clinical efficacy in SLE [34, 35]. For our in vivo experiments, we selected EP that had proved to be an effective treatment in a number of autoimmune diseases/models [14]. EP could protect cells from reactive oxygen damage, down-regulate the expression of NF-kB and decrease the secretion of pro-inflammatory cytokines by inhibiting HMGB1 [36]. Several studies have confirmed that EP is a potent inhibitor of HMGB1 and represents promising intervention in chronic colitis [37]. However, the effectiveness of treating SLE by targeting HMGB1 remained controversial. Anti-HMGB1 antibody ameliorated albuminuria in MRL/lpr mice [13]. Moreover, Zhang et al. demonstrated the effectiveness of the anti-HMGB1 antibody for lupus in BXSB mice [38]. Nevertheless, other study indicated that blocking of HMGB1 by the neutralizing antibody did not affect lupus nephritis in MRL/lpr mice [39]. It was particularly gratifying that administration of EP to inhibit HMGB1 could significantly decrease expression levels of CTLA-4, Foxp3, and IL-10 production after burns, regulating Treg immune [40]. Our results revealed that 100mg/ kg-EP intraperitoneal injection improved Treg immune regulation and reversed the senescence of BM-MSCs via inhibiting TLR4/ NF-κB signaling pathway.

Taken together, our data showed that HMGB1 in SLE bone marrow inflammatory microenvironment could promote the senescence of MSCs via TLR4/NF-κB signaling pathway, and inhibiting HMGB1 by EP could improve lupus nephritis and reverse the senescence signs. Taken with the current data, HMGB1 blockade might be a potential target for treating human SLE.

## MATERIALS AND METHODS

### Patients

Twelve SLE patients between 13 and 43 years of age (mean 28.25±9.69 years) were enrolled in this study. The SLE diagnosis was made based on the criteria proposed by the American College of Rheumatology (2009). The Systemic Lupus Erythematosus Disease Activity Index (SLEDAI) was used to measure the disease activity. All patients were categorized as active using a cut off SLEDAI score of eight. Twelve healthy subjects were used as the normal group. All research subjects were females with a similar age distribution (mean 26.58±7.97 years). All participators gave consent to the study, which was approved by the Ethics Committee of the Affiliated Hospital of Nantong University.

### Proteomics

Bone marrow supernatant samples were analyzed using the PEA and the Olink Immuno-oncology panel (Olink Bioscience AB). This panel includes 92 proteins. The protein analysis is reported as normalized protein expression levels (NPX), which are Ct values normalized by the subtraction of values for extension control, as well as interplate control; the scale is shifted using a correction factor (normal background noise) and reported in log2 scale.

### Isolation, cell culture and identification of BM-MSCs from SLE and normal subjects

MSCs were isolated and expanded from iliac crest BM of all the SLE patients and normal subjects. Five milliliters of heparinized BM were mixed with an equal volume of phosphate-buffered saline (PBS) (Gibco, USA). Then, the resuspended cells were layered over Ficoll solution (1.077 g/mL) and centrifuged at 1,000×g for 30 min at 24 °C. The mononuclear cells were collected at the interface and resuspended in low-glucose Dulbecco Modified Eagle Medium (L-DMEM) (Gibco, USA) supplemented with 10% heat inactivated fetal bovine serum (FBS) (Gibco, USA). Then, the cells were plated at a density of 2×10^7^ cells per 25 cm^2^ dish and cultured at 37 °C in a 5% CO_2_ incubator, and the medium was replaced and non-adherent cells were removed after 5 days and every three days thereafter. When the MSCs became nearly confluent, the adherent cells were released from the dishes with 0.25% trypsin (Gibco, USA), and then replanted at a density of 1×10^6^ cells per 25 cm^2^ dish.

### Western blotting

Cells were washed twice with cold-buffered PBS and were then lysed in RIPA buffer. Equal amounts of protein were separated by 10% SDS polyacrylamide gel electrophoresis (PAGE) and electrophoretically transferred to polyvinylidene difluoride (PDVF) membranes. Membranes were incubated with primary antibodies (1:500) at 4 °C overnight and incubated with horseradish peroxidase conjugated mouse anti-rabbit secondary antibody for 2 h at room temperature. The blots were developed using an enhanced chemiluminescence kit. The following primary antibodies were used: TLR4 (anti-rabbit, Santa Cruz), GAPDH (anti-rabbit, Santa Cruz), β-actin(anti-rabbit, Sangon Biotech), p-IRAK1 (anti- rabbit, Santa Cruz), p-p65 (anti-mouse, Santa Cruz), p65 (anti-rabbit, Santa Cruz), IκBa (anti-rabbit, Santa Cruz), p53 (anti-mouse, Santa Cruz), p27 (anti-mouse, Sangon Biotech).

### SA-β-gal assay

The SA-β-gal activity was determined using a kit from the Chemical Company following the manufacturer’s instructions(Beyotime, China). MSCs were passaged into the 6-well culture plates at a density of 5×10^4^ cells per well for 72 h. Then cells were washed twice with PBS and fixed with fixing solution for 15 min. After incubation with staining SA-β-gal detection solution at 37°C without CO_2_ overnight, the slips were washed and analyzed under the microscope.

### Immunofluorescence assay of the skeleton of MSCs

MSCs were washed twice with PBS and fixed in 4% PFA for 1 h. After permeabilization and bloking, they were incubated with fluorescein isothiocyanate-conjugated phalloidin(Thermo Fisher, Waltham, USA). The stained cells were then examined by a Zeiss Confocal Laser Scanning Microscope.

### Flow cytometry

For cell cycle analysis, MSCs collected and fixed with 70% ethanol at -20°C for 24 h. After being washed with PBS and then treated with 100 μg/ml RNase (Sigma, USA) for 20 min, cells stained with 50 μg/ml propidium iodide (PI) solution (Sigma, USA) for 20 minutes and analyzed by the flow cytometry machine (FACS Calibur, BD Biosciences, USA). The fraction of cells in the G0/G1, S, and G2/M phases were quantified with the ModFit LT system.

For Treg cell analysis, Cells obtained from lymph nodes, spleen and peripheral blood were blocked with anti-CD16/32 and stained with monoclonal anti-mouse CD4 antibody (eBiosciences; San Diego, CA, USA) to assess T cell percentages. Treg cells were characterized by staining for CD4, CD25 and Foxp3, according to the manufacturer’s instructions (Mouse Treg Staining Kit, eBiosciences). All data were acquired in a FACSCalibur flow cytometer (BD Biosciences Immunocytometry Systems, San Jose, CA, USA) and analysed using FlowJo X 10.0.7 software.

### Elisa

Use a serum separator tube and allow samples to clot for 30 minutes before centrifugation for 15 minutes at 1000g. Aspirate serum and assay immediately or aliquot and store samples at ≤ −20 °C. Bone marrow supernatant and serum HMGB1 levels were measured using a commercially available ELISA kit according to the manufacturer’s instructions (Arigo Biolaboratories, USA)

### Cell transfection

BM-MSCs were seeded 2.5 x 10^5^ cells in 6-cm dish with MEM-α medium containing 10% FBS (Gibco) for one day. Transient transfection was performed using the transfection reagent Lipofectamine 2000 (Invitrogen) according to the manufacturer's protocol. The MSCs were then transfected with TLR4 small interfering RNA (siRNA) (GenePharma Co. Ltd.) and a non-specific control siRNA. SiRNA was mixed with Lipofectamine transfection reagent in serum-free medium according to the manufacturer’s instructions.

### Mice and treatments

8-week-old female MRL/lpr mice were obtained from Shanghai SLAC Laboratory Animal Institute Co. Ltd. MRL/lpr mice (n=30) and BALB/c mice (n=10) were maintained in a specific pathogen-free animal facility of the Experimental Animal Center in Nantong University. The animal experimentation was approved by Ethics Committee of Nantong University. The mice were intraperitoneally injected with EP (40 mg/kg or 100 mg/kg) or vehicle control (saline) three times per week. MRL/lpr mice were divided into three groups. All animal procedures were approved by the institute’s Institutional Animal Care and Use Committee.

### Isolation, culture and identification of BM-MSCs from MRL/lpr mice

The BM was flushed out of the femurs and tibias removed from MRL/lpr and BALB/c mice using 10 ml PBS with 100 U/ml heparin in a syringe. The cells were centrifuged at 1000 ×g for 10 min. The cell pellet was resuspended in 5 ml L-DMEM supplemented with 10% FBS (Gibco, USA) and plated in a 25 cm2 dish to allow the MSCs to adhere. Cultures were maintained in a humidified atmosphere with 5% CO2 at 37°C.

### Albuminuria

24h urine samples were collected from each mouse by metabolic cages method once every two weeks. Urinary albumin levels were measured using a commercially available ELISA kit (BioAssay Systems, Hayward, CA, USA) according to the manufacturer’s instructions.

### Renal histology studies

At the time of euthanization, kidney specimens were obtained, fixed in 10% formaldehyde and embedded in paraffin. Sections (4 μm thickness) were prepared and then stained with haematoxylin and eosin (H&E). The kidney sections were coded and examined by two independent observers who were blinded to the treatment groups.

### Statistical analysis

All data are presented as the mean±standard deviation (SD) from at least three independent experiments. All statistical analyses were performed using the SPSS 21.0 software, and the data were analyzed using Student’s t test with P<0.05 considered statistically significant.
